# Polytetrafluoroethylene Isolation of the Periodontal Sulcus for Cementation of Full Veneer Restorations Using a Biologically Oriented Preparation Technique (BOPT): An In Vitro Study

**DOI:** 10.3390/jcm14155305

**Published:** 2025-07-27

**Authors:** José Félix Mañes, Federica Tripodi, Jorge Alonso Pérez-Barquero, Blanca Serra-Pastor, Ana Roig-Vanaclocha, Jesús Maneiro-Lojo, Ignazio Loi, Rubén Agustín-Panadero

**Affiliations:** 1 Department of Stomatology, Faculty of Medicine and Dentistry, University of Valencia, 46010 Valencia, Spain; jose.manes@uv.es (J.F.M.); jorgealonso86@gmail.com (J.A.P.-B.); aroigva@hotmail.com (A.R.-V.); susomaneiro@hotmail.com (J.M.-L.); rubenagustinpanadero@gmail.com (R.A.-P.); 2 Private Practice, 46008 Valencia, Spain; fedetripe@gmail.com; 3 Private Practice, 09125 Cagliari, Italy; loi.ig@tiscali.it

**Keywords:** BOPT, PTFE, isolation, cementation, vertical tooth preparation

## Abstract

**Background:** Prosthetic cementation using the biologically oriented preparation technique (BOPT) presents challenges in removing excess cement from the gingival sulcus, due to the absence of a finishing line and the impossibility of using absolute isolation with a rubber dam. This study aimed to evaluate the effectiveness of relative isolation using polytetrafluoroethylene (PTFE) tape in reducing cement retention during BOPT cementation. **Methods:** Fifteen 3D-printed resin models were created from an intraoral scan of a patient restored with BOPT in both upper central incisors. Each model included removable gingiva. Splinted polymethylmethacrylate (PMMA) provisional crowns were fabricated and cemented with temporary cement. One central incisor was isolated with PTFE (0.1 mm or 0.2 mm), while the contralateral tooth was left unisolated as a control. After debonding, digital scanning and volumetric analysis using root mean square (RMS) deviation were performed to quantify retained cement. Paired *t*-tests were applied to compare groups. **Results:** The mean RMS for the PTFE group was 0.1248 ± 0.0519 mm, compared to 0.1973 ± 0.0361 mm in the non-isolated group (*p* < 0.001). No significant difference was found between PTFE thicknesses of 0.1 mm and 0.2 mm (*p* = 0.388). **Conclusions:** PTFE tape is effective for relative isolation when rubber dam placement is not feasible in BOPT restorations. Further clinical studies are recommended to confirm these findings in vivo.

## 1. Introduction

Adhesive cementation is a technically complex process due to the large number of influencing factors. Absolute isolation with a dental dam is recommended for adhesive cementation procedures to minimize moisture and facilitate the removal of cement debris [1,2]. The biologically oriented preparation technique (BOPT) presents a challenge for rubber dam isolation due to the lack of a finishing line, the presence of a converging axial wall morphology, and the marginal restoration–preparation fit being located inside the gingival sulcus [3,4,5,6,7].

As an alternative to the conventional use of a rubber dam, polytetrafluoroethylene (PTFE) tape, also known as Teflon, is proposed as a material that exhibits a high degree of surface conformity and can be manipulated without readily tearing [8].

In 2013, Loi presented a novel preparation technique that represented a paradigm shift in the field of fixed restorations. This technique has been shown to have numerous advantages [9], but it is important to note that it also has disadvantages, including the difficulty of removing excess cementing material [10]. Preparations with the BOPT are subgingival, and since they do not have a finishing line and cannot be isolated, there is a high risk of invasion of the supracrestal tissue attachment by excess cement. This excess cement can be detrimental to the periodontium and compromise the optimal adaptation of the adjacent gingival tissues, a hallmark of the BOPT [11]. Consequently, the biological outcomes associated with this treatment are highly dependent on the operator’s expertise and are related to the skill of the dentist performing the technique. Additionally, the selection of reconstruction materials was constrained due to the necessity of addressing the humidity present in the oral cavity and the crevicular sulcus. This limitation compelled the utilization of less humid-sensitive cements, such as glass ionomer cements. Prior to the advent of relative isolation with PTFE, BOPT restorations employed exclusively metal-ceramic and zirconia materials, as they did not necessitate adhesive cementation for optimal mechanical behavior [12,13]. However, the arrival of PTFE has led to significant advancements in the field, allowing the utilization of high-strength feldspathic ceramics, such as those reinforced with lithium disilicate, ensuring optimal adhesion.

The clinical significance of this study lies in validating the technique described by Roig-Vanaclocha [12] through an in vitro study. The study will analyze the difference between the amount of cement remaining in the sulcus in a tooth isolated with PTFE and another tooth without PTFE. It will also compare different thicknesses of PTFE to evaluate clinical differences in its use.

The objective of this study was to evaluate the effect of PTFE tape isolation during the temporary cementation of BOPT-prepared abutments, compared to no isolation.

The null hypothesis was that there would be no statistically significant difference in the amount of cement remaining in the sulcus between BOPT preparations cemented with and without PTFE isolation.

## 2. Materials and Methods

The experimental model was obtained from a real case treated with the BOPT on the two upper central incisors. This research was conducted in accordance with the principles outlined in the Declaration of Helsinki (1975, revised in 2013). Although the present study was conducted in vitro, an intraoral scan from a patient was used for the subsequent fabrication of study models. The patient provided informed consent for the use of their intraoral scan as study material. This research was approved by the Ethics Committee of the University of Valencia, under registration number 2024-ODON-3325009.

For the registration of the patient, and with the intention of creating a digital model in standard tessellation language (STL) format with an uncollapsed periodontal sulcus, the triple scan technique described by Agustín-Panadero was used [10]. The initial scan captured the patient’s upper arch, including both upper central incisors that had undergone BOPT preparation and their cemented provisional prostheses. The subsequent scan was also of the upper jaw and captured the teeth that had been prepared without a provisional prosthesis. The final scan captured the out-of-mouth provisional prosthesis, which was fabricated from polymethylmethacrylate (PMMA). These three files were aligned in Geomagic reverse engineering software (3D Systems), and the Boolean algorithm was run to create an STL master model. The periodontal sulcus adjacent to the prepared teeth was open, uncollapsed, and completely adjusted to the cervical emergences of the provisional prosthesis.

**Figure 1:**
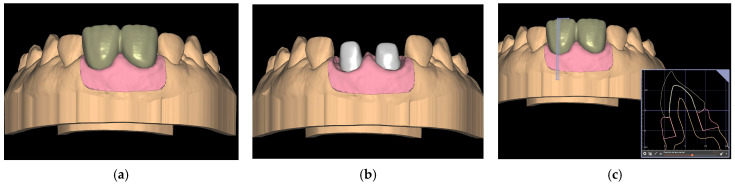
The design of the models incorporates removable gingivae and splinted provisional prostheses. Figure (**a**) shows the digital model with the provisional. Figure (**b**) shows the digital model with the tooth preparations and figure (**c**) shows the sagittal section of the digital model.

Subsequently, utilizing Exocad software (Exocad GmbH; DentalCAD 3.1 Rijeka), 15 models were designed with removable gingivae and 15 splinted provisional prostheses, thereby restoring both upper central incisors with a cervical angulation of 60° (Figure 1).

Following the conception of the designs, the models were exported in STL format and printed with the Formlabs 3B printer (Formlabs). The company’s proprietary laminating software, Preform (PreForm. version 3.34.3), was utilized for this purpose.

**Figure 2:**
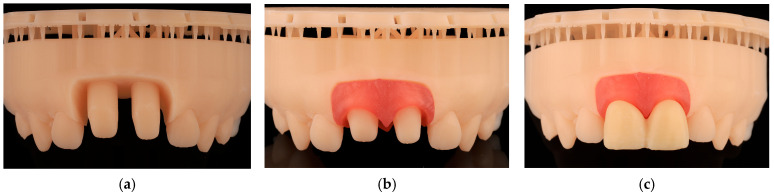
Resin model, gingiva and provisional prosthesis. (**a**) Digital printed model of tooth preparations without removable gingiva. (**b**) Digital printed model of tooth preparations with removable gingiva. (**c**) Digital printed model of tooth preparations with provisional restorations.

The models were printed with Model 1L V3 resin (Formlabs), the gingivae with Flexible 80a resin (Formlabs), and the provisional protheses with Temporary crown and bridge color A2 resin (Formlabs). The washing process was performed with the Form Wash machine (Formlabs), and the final curing was executed with the Form Cure machine (Formlabs) (Figure 2).

**Figure 3:**
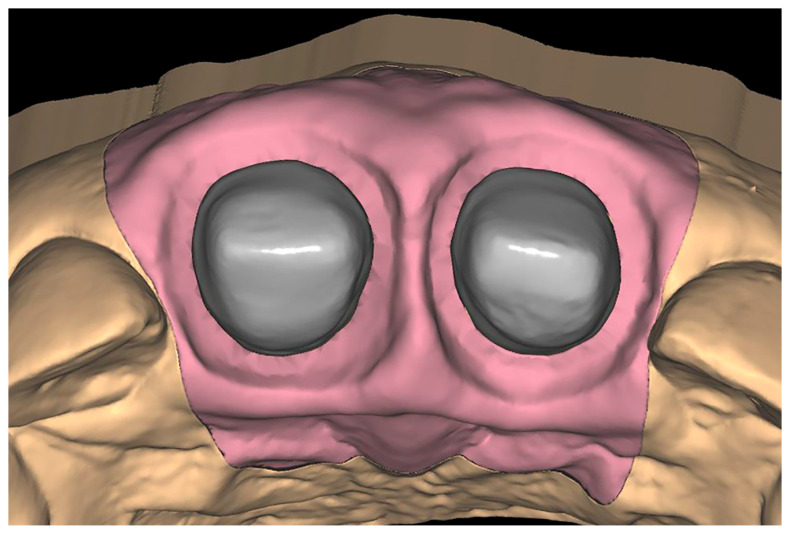
Scanning of the model.

After the 15 models were printed, they were scanned with their corresponding gingivae mounted using the 3 Shape Trios scanner (3shape) (Figure 3).

**Figure 4:**
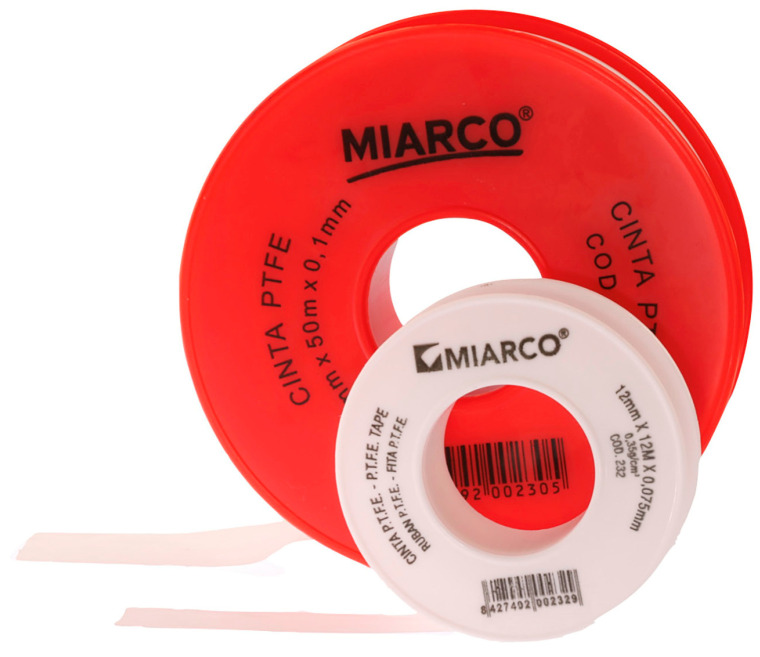
PTFE tape of two different thicknesses: 0.1 mm and 0.2 mm.

**Figure 5:**
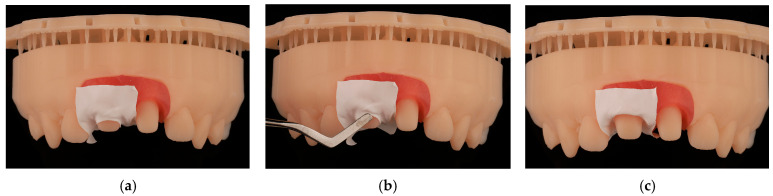
PTFE tape adapted to the sulcus of tooth 1.1, while tooth 2.1 was left untouched. (**a**) Starting the adaptation of the PTFE tape in the preparation. (**b**) The PTFE was then placed inside the sulcus with the help of a composite spatula. (**c**) Final adaptation of the PTFE tape in the sulcus.

**Figure 6:**
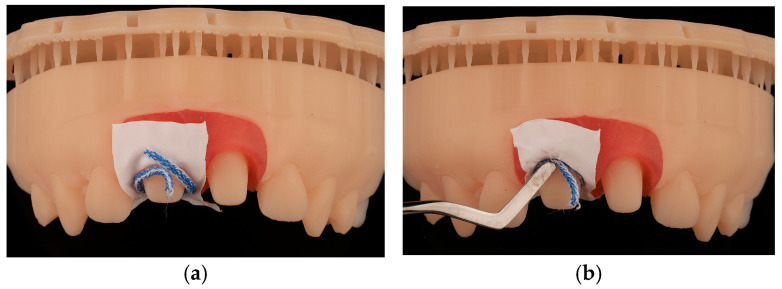
Placement of retraction cord to adapt the Teflon tape to the inner wall of the gingival sulcus. (**a**) Starting the insertion of the retraction cord. (**b**) Using a specific instrument, the cord was inserted which enabled the adaptation of the Teflon tape to the internal wall of the gingival sulcus.

**Figure 7:**
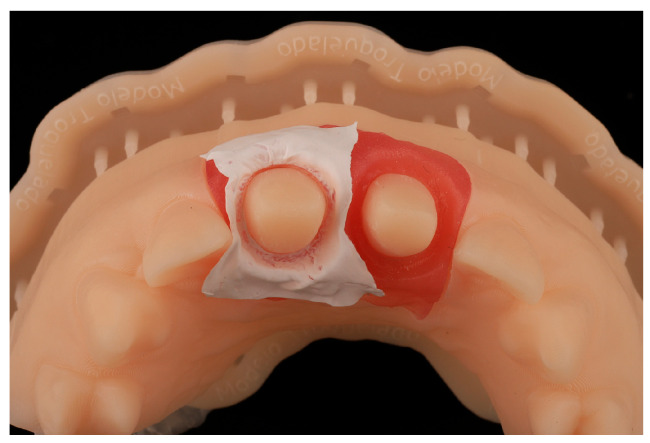
Isolation of the mesial, buccal, lingual, and distal tooth surfaces of the upper right central incisor.

For this study, PTFE tape (S&M) of two different thicknesses was used: 0.1 mm and 0.2 mm (Figure 4). The protocol^8^ was followed, with 2 cm of the Teflon tape being cut and a perforation created using an Ivory rubber dam perforator (BADER) in the XS size. The PTFE was then placed inside the sulcus without contacting the adjacent tooth (Figure 5) with the help of a composite spatula. Using a specific instrument (Fischer Ultrapack Packer), a retraction cord was inserted (Ultrapack, size 1), which enabled the adaptation of the Teflon tape to the internal wall of the gingival sulcus (Figure 6). This approach facilitated the isolation of the mesial, buccal, lingual, and distal tooth surfaces of the upper right central incisor (Figure 7). Given the presence of two abutments, it was decided to apply Teflon to only one of them, with the other tooth (the upper left central incisor) serving as a control group without Teflon.

**Figure 8:**
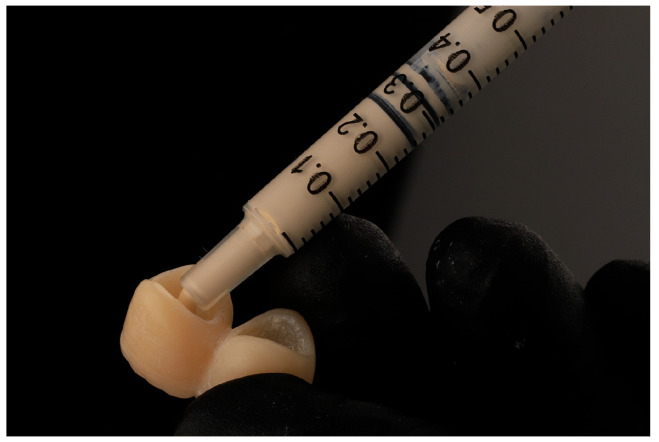
Syringe with Tempbond temporary cement.

**Figure 9:**
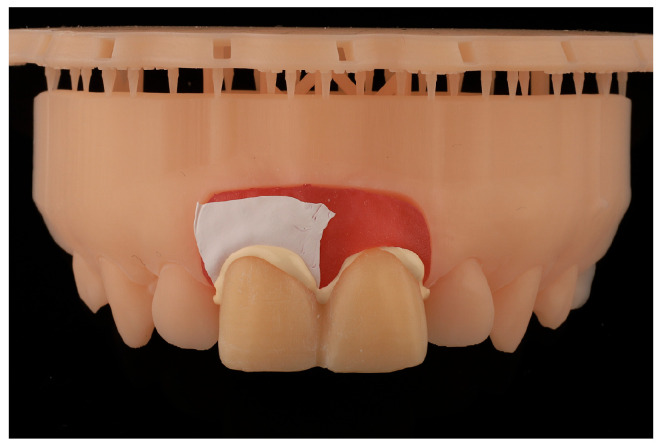
Placement of the temporary crowns with cement on the abutments.

Following the adaptation of the Teflon, the cementing procedure was executed by the same operator (F.T.). The Tempbond Temporary Cement Type-1 (Kerr) was utilized with a syringe to ensure the uniform application of cement in both crowns. A plastic syringe without a needle was employed to administer 0.05 mL of cement into each crown (Figure 8) and, subsequently, the crowns were mounted on the abutments (Figure 9). Prior to the complete setting of the cement, the Teflon was meticulously removed by the same operator (F.T.), to detach the extravasated cement from the gingival sulcus. This procedure was executed with utmost care, ensuring the integrity of the Teflon tape, which was gently stretched along one of its corners to prevent tearing.

Subsequent to the removal of the tape, equal and constant pressure on both abutments was ensured by applying force using a standardized weight (1 kg). Prior to cementation, a 1.5 cm resin plate was fabricated. One side of the plate embraced the incisal part of the crowns, while the other side formed a flat surface for the placement of the weight.

Following the completion of the six-minute setting period as instructed by the manufacturer, the plate and weight were removed, and the residual excess was meticulously removed from the cemented crowns using a 1086/11 exploration probe (Carl Martin). The operator was allotted 30 s per crown to ensure the removal of excess cement, thereby standardizing the cleaning process.

**Figure 10:**
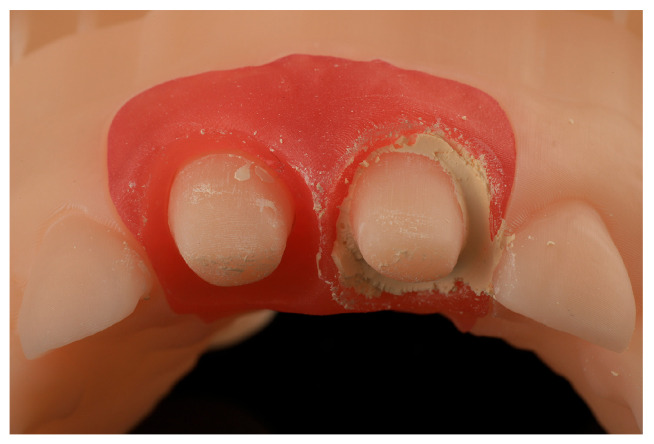
Vestibular and incisal view of the sulcus: 1.1 (with Teflon) and 2.1 (without Teflon).

The temporary crowns were then debonded using an upper incisor forceps (Carl Martin) (Figure 10). For the quantification of cement remaining in the sulcus, the experimental models were scanned and saved in STL format by a different operator (J.F.M.); so that the measurements were performed by a blind operator.

Following the initial cementation process with 0.1 mm PTFE tape, the procedure was repeated with 0.2 mm PTFE tape. To this end, following the cementation sequence with the 0.1 mm PTFE tape, the models were cleansed with orange solvent spray (HAGER WERKEN). This step ensured that the entire procedure could be repeated from the beginning with the 0.2 mm thickness tape, using a pristine model.

**Figure 11:**
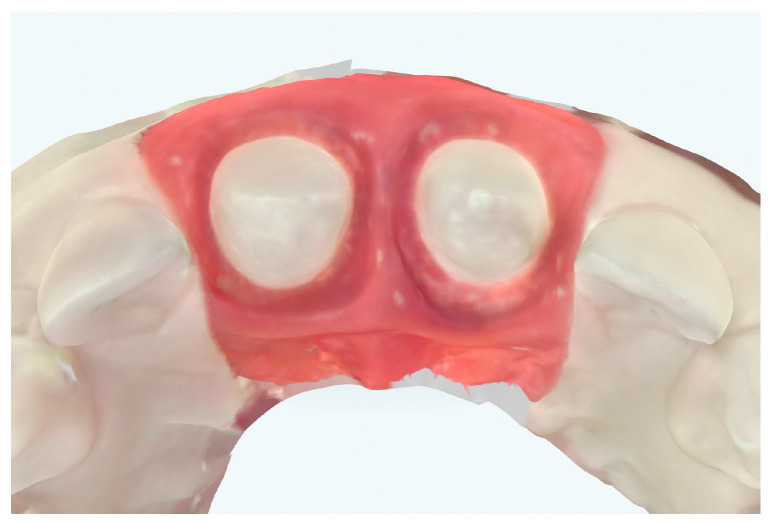
Scanning of the initial model. The STL1 file corresponds to the model prior to cementation.

**Figure 12:**
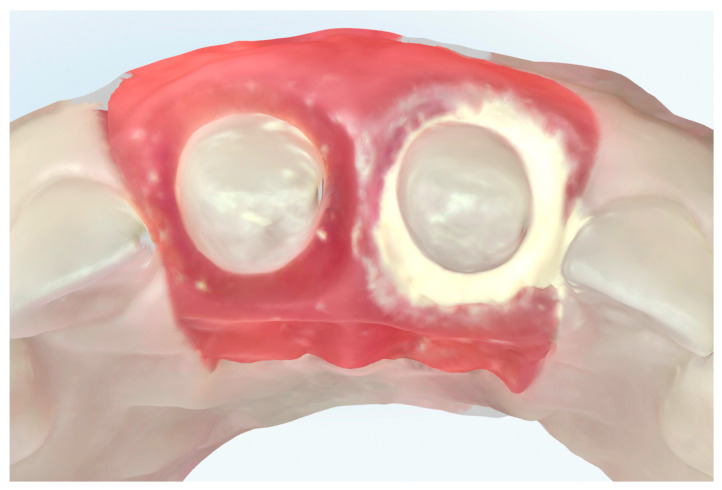
Scanning after cementation. The STL2 file corresponds to the model with cement that used the 0.1 mm PTFE tape and the control group.

Subsequent to the cementation protocol, three scanned files were obtained for each model, which were designated as follows: STL1—this file corresponds to the model that was not cemented (Figure 11); STL2—this file corresponds to the model that was cemented using 0.1 mm PTFE tape and the control group (Figure 12); STL3—this file corresponds to the model that was cemented using 0.2 mm PTFE tape and the control group.

**Figure 13:**
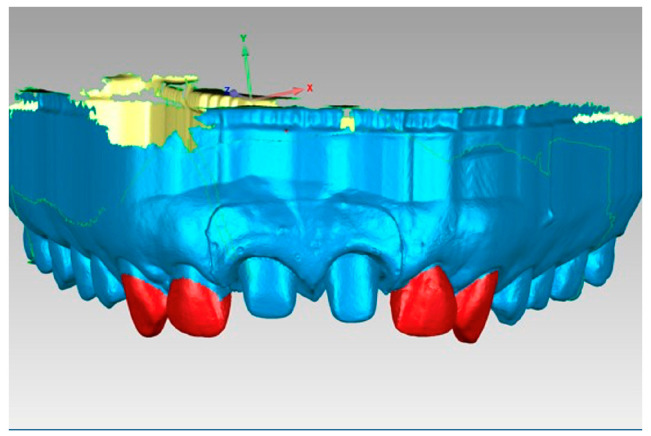
Image of the area selected to run the best fit algorithm.

The amount of cement remaining as a function of the cementing protocol was then analyzed using Geomagic reverse engineering software (Geomagic Design X Go). Initially, the 15 STL1 files were aligned using the best fit algorithm, so that all the uncemented models would be in the same spatial position. Subsequently, the STL2 and STL3 files were aligned to the corresponding STL1 using the best fit algorithm. For this purpose, the common areas of the teeth adjacent to the treated teeth were selected, i.e., the teeth in position 13, 12, 22, and 23, since they are teeth that have not undergone any variation between models (Figure 13).

**Figure 14:**
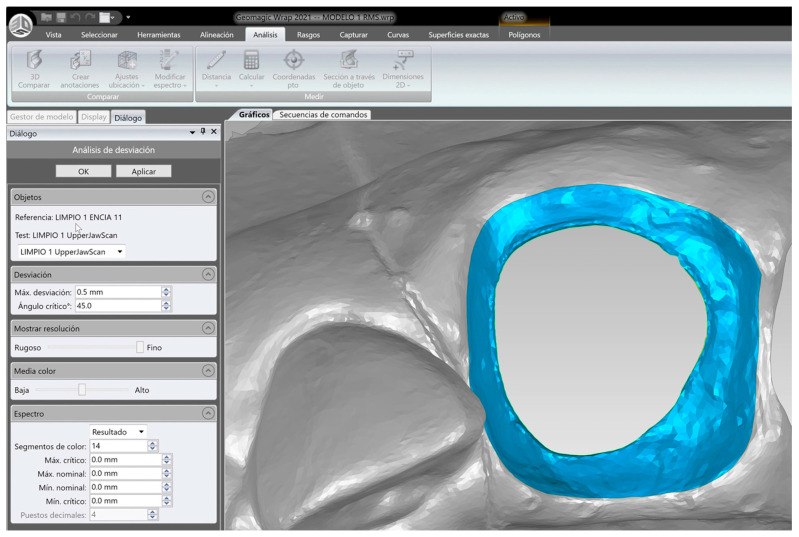
Details of the generation of the new mesh corresponding to the gingival sulcus area. The union of the external and internal curves is drawn in the software.

Once all the models were aligned, two polygonal curves were created using the ”draw curve” function. The first curve was positioned in the inner part of the sulcus, in contact with the tooth, while the second curve was placed in the outer part of the sulcus, where the emergence of the provisional restoration ends. These curves were then transferred to models SL1, 2, and 3, and the ”trim with curve” command was executed. This process yielded two independent meshes per model, STL1_11 and STL1_21 (Figure 14).

**Figure 15:**
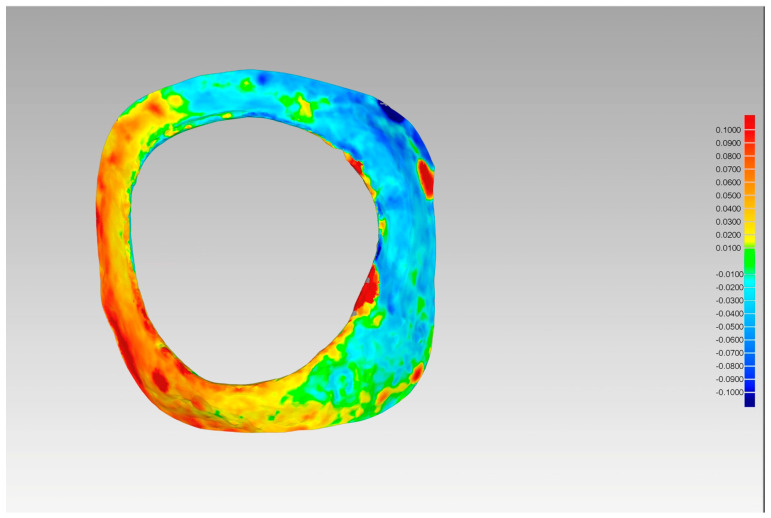
Color map with RMS values.

Following the generation of new meshes in each of the models, a comparative analysis was conducted between STL1_11 and STL2_11 and STL3_11, as well as between STL1_21 and STL2_21 and STL3_21. The root mean square (RMS) method was employed to ascertain the quantity of cement remaining, contingent upon the cementing protocol (Figure 15).

A comprehensive descriptive and inferential analysis was conducted, providing the most relevant statistical data for the quantity variable in each of the designated situations (with/without Teflon, thickness 0.1 mm/0.2 mm): mean, standard deviation, minimum, maximum, 25th percentile, median, and 75th percentile. The fit to normal distribution of the quantity measurement was checked by Kolmogorov–Smirnov test for the sample of 30 observations. This was followed by the Shapiro–Wilk test for the subsamples based on thickness, thereby confirming the validity of the underlying assumptions. The analysis approach was rooted in parametric methods, with the t-student test for dependent samples (paired *t*-test) being implemented to make comparisons between the mean values of the quantity across the Teflon and non-Teflon groups, as well as between the two thicknesses that were utilized. The significance level used was 5% (α = 0.05). The dependent samples t-test achieved a power of 93% to detect a mean difference in retained cement between groups compatible with a mean effect size (d = 0.5) for 95% confidence. For comparisons between the two thickness levels, the power decreases to 43.7% under the same conditions, which is acknowledged as a limitation. However, when considering a large effect size (d = 0.8), which is more relevant from a clinical perspective, power increased to 82.1%, thereby supporting the validity of the observed results.

## 3. Results

Prior to the statistical analysis of the influence of PTFE isolation on cementation outcomes, the experimental groups were compared to confirm homogeneity of baseline conditions. No significant differences were observed between control conditions (t = 0.11; *p* = 0.911), confirming that the samples were comparable.

**Figure 16:**
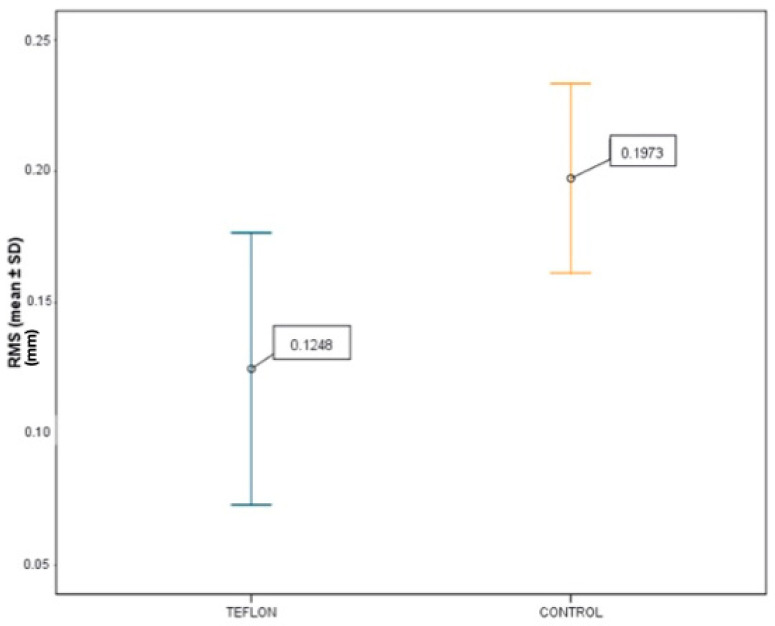
Lower cement retention in the Teflon group (RMS 0.1248 mm) compared to the control group (RMS 0.1973 mm).

The comparison between the PTFE-isolated group and the control group (no isolation) revealed a mean RMS value of 0.1248 ± 0.0519 mm for the PTFE group, and 0.1973 ± 0.0361 mm for the control group (Figure 16). The paired t-test indicated a statistically significant difference (*p* < 0.001), with a 95% confidence interval for the mean difference ranging from −0.0902 mm to −0.0550 mm. This result supports the conclusion that the use of PTFE significantly reduces cement retention during BOPT cementation procedures.

**Figure 17:**
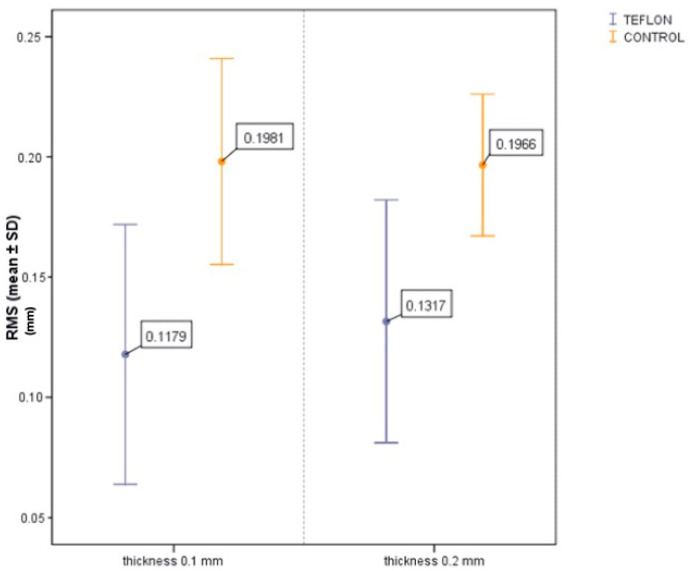
There are no significant differences between the two PTFE thicknesses.

Regarding the comparison between PTFE thicknesses, the RMS was 0.1179 ± 0.0540 mm for the 0.1 mm tape and 0.1317 ± 0.0505 mm for the 0.2 mm tape. The difference between the two was not statistically significant (*p* = 0.388), and the 95% confidence interval for the mean difference (0.1 mm–0.2 mm) ranged from −0.0468 mm to 0.0193 mm (Figure 17). This interval includes zero, further indicating the absence of a meaningful difference between the two thicknesses in terms of cement removal efficacy.

## 4. Discussion

As stated in the literature, the extravasation of resin cement into the gingival sulcus [14,15,16,17,18] during the cementation of BOPT restorations may lead to inflammation and degradation of periodontal tissues over time. This risk is particularly concerning in the context of BOPT, a technique that lacks a definitive horizontal finish line and therefore complicates the removal of excess cement [4,5,6,7,10,11,12]. The vertical, convergent nature of the preparation precludes the use of rubber dam isolation with clamps, as its stabilization is technically unfeasible [4,5,6,7,10,11,12].

In this context, relative isolation using PTFE tape emerges as a practical alternative. Unlike the rubber dam, which offers complete moisture control but is inapplicable in subgingival BOPT preparations, PTFE allows for flexible, localized isolation. While it does not eliminate moisture entirely, it creates a barrier that can significantly reduce cement extrusion into the sulcus and facilitate cleaner margins. Compared to absolute isolation, this relative method balances clinical feasibility with meaningful reduction in cement-related complications.

The present in vitro study supports the clinical utility of PTFE in improving cement control during temporary cementation of BOPT restorations. The statistically significant reduction in retained cement (*p* < 0.001) with PTFE isolation underscores its effectiveness. Although the comparison between PTFE thicknesses (0.1 mm vs. 0.2 mm) did not yield a statistically significant difference (*p* = 0.388), the results indicate that 0.1 mm tape may be preferred due to its better adaptability and easier placement in subgingival areas.

The effectiveness of polytetrafluoroethylene (PTFE) tape in subgingival cementation has been increasingly validated by recent studies, supporting the results of the present investigation. In particular, Djordjević et al. demonstrated that the amount of residual cement significantly varies according to the cementation technique and the subgingival localization of the crown-abutment margin. Their study reported that standard techniques resulted in significantly more excess cement than PTFE-based, especially when the finish line was placed 1.5 mm or 3 mm below the gingival margin [19]. This reinforces the relevance of using PTFE in clinical scenarios where access to subgingival zones is limited, particularly in BOPT-type preparations.

Furthermore, Schuh et al. introduced the ”Teflon tape technique” as an efficient and practical method for improving field isolation during adhesive procedures. They emphasized the tape’s adaptability and its capacity to ensure excellent moisture control while preserving access to subgingival areas. This technique was presented as a suitable alternative to rubber dams [20]. Their findings align with our conclusion that PTFE tape is a valuable resource for clinicians seeking predictable isolation in cases where conventional means are either ineffective or contraindicated.

There are some studies in the literature [8,21] reporting that PTFE tape significantly decreases the presence of residual cement in implant-supported prostheses, compared to other isolation techniques such as rubber dams or the use of dental probes. This effectiveness was especially notable when the prosthetic margins were subgingival, supporting its use in techniques like BOPT, where finish lines are intentionally omitted for biological contouring.

Additionally, Thimmappa et al. compared various non-invasive gingival displacement systems and found Merocel strips to be highly effective in achieving gingival retraction. However, they did not address cement residue management, which remains a significant limitation when compared to the PTFE technique [22].

Collectively, these studies underscore the importance of selecting isolation and cementation techniques that minimize the risk of residual cement, particularly in subgingival regions where access and visibility are limited. The results of the present study confirm that PTFE tape offers a viable compromise between clinical efficiency and biological safety, especially when absolute isolation is unfeasible. Nonetheless, further in vivo studies are necessary to evaluate long-term effects on gingival health, marginal integrity, and restoration longevity.

Regarding the prosthetic design, all crowns in this study were splinted. This methodological decision was made to standardize the cementation process and ensure that the pressure applied during crown seating was consistent across all samples. Splinting eliminated variability in insertion force or angulation, which could otherwise influence cement extrusion and retention. While this design does not fully replicate individual clinical crowns, it was necessary to control for mechanical variables during the experimental procedure.

From a technical standpoint, the PTFE isolation method is relatively easy to implement but does require precise manipulation to avoid dislodgment during seating. The tape must be carefully adapted to the sulcus without causing trauma or affecting the fit of the crown. Clinicians should also be aware of the limitations of temporary cements, which may behave differently from permanent resin cements in terms of flow, setting, and cleanup.

To the best of our knowledge, this is the first study to evaluate the volumetric effect of PTFE isolation on cement retention in BOPT restorations using 3D analysis. While promising, these results should be interpreted within the constraints of an in vitro setting. Similar methodological approaches have been reported in the recent literature. For example, in 2022, Kurian described a modified PTFE isolation technique using a 0.1 mm PTFE tape and dental floss to minimize residual cement under the pontic during the cementation of fixed partial dentures [13]. Additionally, professional engineering software, such as Geomagic, also employed in our study, has been widely used for volumetric quantification and trueness analysis in dental research [23]. Moreover, the use of RMS (root mean square) as a metric for assessing 3D discrepancies is well documented. In 2019, Papaspyridakos et al. applied RMS analysis to evaluate the accuracy of digital workflows and 3D-printed full-arch implant models [24].

It is important to acknowledge the limitations inherent to this experimental design. The model did not simulate gingival fluid, plaque, saliva, or the dynamic nature of intraoral temperature and humidity. These factors could influence cement flow and setting time in clinical conditions. Therefore, while the in vitro results are promising, further clinical studies are necessary to confirm their applicability.

Finally, while this study focused on temporary cementation for methodological reasons, allowing crown retrieval and precise volumetric analysis, PTFE isolation may also be relevant in the context of permanent cementation. Residual resin-based cements can provoke a stronger inflammatory response than provisional ones; thus, effective isolation becomes even more critical in definitive procedures.

## 5. Conclusions

- PTFE tape is an effective alternative for relative isolation during BOPT cementation, significantly reducing excess cement in the gingival sulcus compared to no isolation.
- A 0.1 mm PTFE thickness performs similarly to 0.2 mm in terms of cement control, with the added benefit of better subgingival adaptability and easier handling.
- Rubber dam isolation is not feasible in BOPT preparations due to the convergent, subgingival design; PTFE offers a practical solution in these cases.
- All crowns in this study were splinted to ensure consistent seating pressure, eliminating variability in cementation force and insertion angle.
- The PTFE isolation technique is clinically applicable, but it requires careful manipulation to avoid dislodgement and ensure precise adaptation to the sulcus.
- While the study focused on temporary cementation, the findings are also relevant for permanent cementation, where excess resin cement poses an even greater risk for periodontal inflammation.
- Further clinical studies are needed to confirm these findings under intraoral conditions, including the presence of saliva, gingival fluid, temperature fluctuations, and plaque.

## Data Availability

The original contributions presented in this study are included in the article. Further inquiries can be directed to the corresponding author(s).
